# 

**Published:** 1874-10

**Authors:** 


### CONTENTS:

COMMUNICATIONS:

Progressive and Retrograde Metamorphosis of Bones and Teeth ... 405
Offensive breath from Artificial Dentures ... 411
Why not? ... 415
Improvements in Working Rubber ... 417

PROCEEDINGS OF SOCIETIES:

American Dental Association ... 389
South Carolina Dental Association ... 430
Annual Meeting Minnesota Dental Association ... 433
American Dental Society of Europe ... 437

EDITORIAL:

Diversity of Experience in Dental Practice ... 440
The Dental Colleges ... 441
Treatment of Diseased Gums ... 443
Vulcanite Litigation ... 445
Notice ... 447
Twelfth Annual Meeting of the Connecticut Valley Dental Society ... 448

